# Protective effect of *Berberis vulgaris* on Fenton reaction-induced DNA cleavage

**Published:** 2019

**Authors:** Nooshin Sadat Asadi, Mohammad Mehdi Heidari, Mehri Khatami

**Affiliations:** 1 *Department of Biology, Faculty of Science, Yazd University, Yazd, Iran*

**Keywords:** Antioxidant activity, Berberis vulgaris, DNA damage, Fenton reaction

## Abstract

**Objective:**

*Berberis vulgaris* contains antioxidants that can inhibit DNA cleavage. The purpose of this study was to evaluate the antioxidant and protective activity of *B. vulgaris* on DNA cleavage.

**Materials and Methods:**

In this study, the antioxidant capacity of *B. vulgaris *was investigated using DPPH and its protective effect was evaluated on *pBR322* plasmid and lymphocyte genomic DNA cleavage induced by Fenton reaction, by DNA electrophoresis.

**Results:**

Aqueous extract of *B. vulgaris* presented dual behavior with a potent antioxidant activity at 0.25and 0.75mg/ml for pBR322 plasmid and lymphocyte genomic DNA, respectively, but a pro-oxidant activity was observed at higher concentrations.

**Conclusion:**

Our results indicated that *B. vulgaris* extract an inhibit Fenton reaction-induced DNA cleavage and oxidative cleavage of double-stranded DNA assay is a powerful technique that can be used to determine the antioxidant and pro-oxidant properties of a compound on cellular components such as DNA.

## Introduction

Cellular DNA is permanently exposed to oxidative stress and free radicals produced by cellular metabolism and exogenous agents (Adly, 2010 ▶). These free radicals can lead to oxidative DNA damage (Orrenius et al., 2007 ▶). High levels of reactive oxygen species (ROS) play an important role in the development of many human diseases like cancers, cardiovascular diseases, diabetes, atherosclerosis, and neurological disorders (Huang, 2003 ▶; Scheibmeir et al., 2005 ▶; Zhao and Zhao, 2013 ▶). Over the last decades, there has been a huge interest in identifying defense mechanisms that suppress or retard the oxidative DNA damage arising from free radicals or ROS (Ajith, 2010 ▶; Charehsaz et al., 2015 ▶). The antioxidants, abundant in fruit and vegetables can impede several types of cancers by interfering with the binding of carcinogens to DNA or through scavenging ROS (Abbas et al., 2014 ▶; Rajendran et al., 2014 ▶).* Berberis vulgaris* (Berberidaceae family) is a fruit rich in antioxidants because of the presence of high levels of phenolic and anthocyanin compounds (Yildiz et al., 2014 ▶). This plant which grows in Europe, North Africa, the Middle East, and central Asia is a spiny shrub with 1–3m height, yellow wood and ovate leaves, bearing pendulous yellow flowers succeeded by oblong red fruits (Mohamadi et al., 2012 ▶; Yildiz et al., 2014 ▶).These fruits have been frequently consumed as food *garniture* in Persian food and in preparing juices, jellies, carbonated drinks, candy, food color powder, jam, marmalade, chocolates, and fruit nectars owing to its mellow taste and color (Joukar and Mahdavi, 2014 ▶; Charehsaz et al., 2015 ▶). Barberry extract contains basic components with antioxidant characteristics such as berberine, berbamine, palmatine, oxyacanthine, malic acid, and berberubin (Mohamadi et al., 2012 ▶). It was proven that it can be used as a therapeutic agent against a number of diseases like hyperlipidemia, diabetes, metabolic syndrome, obesity, and coronary artery disease(Bouayed and Bohn, 2010 ▶). Hydroxyl radical ( OH) can attack different biomolecules including DNA, and play a basic role in the formation of DNA oxidative damage. Hydroxyl radicals can be produced through Fenton reaction induced by H_2_O_2_ and transition metals (Fe^2+^,Cu^2+^) (Goldstein et al., 1993 ▶; Henle and Linn, 1997 ▶).The DNA nicking assay mimics the *in vivo *biological condition and is based on the Fenton reaction with the production of hydroxyl free radicals from intracellular iron. In this assay, formation of ^•^OH during the reaction causes the initial supercoiled configuration of plasmid DNA (Form I) to from supercoiled to open circular (Form II) and nicked linear forms (Form III) that present different mobility properties on gel electrophoresis (Kitts et al., 2000 ▶). Several studies have shown that organic solvents inhibit Fenton’s reaction and prevent DNA strand breaks (Engelmann et al., 2003 ▶; Leba et al., 2014 ▶).

The current study was designed to evaluate the protective effect of *B. vulgaris* juice as a natural antioxidant against Fenton-type oxidative cleavage of double-stranded DNA (Genomic DNA and *pBR322* plasmid).

## Materials and Methods

**Chemicals and reagents**

Hydrogen peroxide (H_2_O_2_), ferrous sulfate(FeSO_4_.7H_2_O), ethylene diamine tetra acetic acid disodium salt dehydrate (EDTA-Na_2_), hydrochloric acid, 1, 1-diphenyl-2-picrylhydrazyl (DPPH), K_2_HPO_4_ and KH_2_PO_4_ were bought from Fluka Sigma–Aldrich (Steinheim, Germany). Agarose, DNA ladder, *pBR322* plasmid DNA were purchased from SinaClonBioScience Co. (Tehran, Iran). All other chemicals and reagents used were of analytical grade.

**Preparation of extract and evaluation of antioxidant activity of** ***B. vulgaris***

Berberis fruits from the South Khorasan province of Iran were collected. The preparation of aqueous extract of Berberis fruit and 1, 1-diphenyl-2-picrylhydrazyl (DPPH) free radical was measured based on Hoshyar et al. study (Hoshyar et al., 2016 ▶). The free-radical-scavenging activity of the extract of Berberis fruit was measured based on Brand-Williams et al. study (Brand-Williams et al., 1995 ▶). DPPH free radical scavenges ability of Berberis is reported as percentage (%).

***B. vulgaris*** **effect on** ***pBR322*** **DNA nicking assay**

The optimal conditions for DNA nicking assay were set based on a previous study (Leba et al., 2014) with some modifications. Here, 1μl of *pBR322* plasmid DNA (25μg/μl) was mixed with phosphate buffer (H_2_PO_4_, 8.3mM, pH 7.4), 5mM of H_2_O_2_ and 0.33mM of FeSO_4_ and 0.62mM of EDTA in a final volume of 20μl and were incubated for 20 min at 37°C. To assess antioxidant capacity of* B. vulgaris* against DNA nicking, various concentrations of *B. vulgaris *extract (0.25, 0.5, 0.75 and 1.0mg/ml) were used. Also, 1μl of *pBR322* plasmid DNA (25μg/μl) was used as DNA protection control.

***B. vulgaris*** **effect on PCR-based genomic DNA nicking assay**

DNA was extracted from healthy blood samples by using a standard DNA extraction kit. A 198-bp fragment of the *MTHFR* gene was amplified using PCR from blood genomic DNA, by using the following primers: 5′-TGAAGGAGAAGGTGTCTGCGGGA-3′ and reverse 5′-AGGACGGTGCGGTGAGAGTG -3′. PCR amplification was carried out at 94ºC for 5min, followed by 30 cycles (denaturation, 30sec at 94°C; annealing, 30sec at 60°C; and extension, 30sec at 72°C) and 1 final extension cycle at 72°C for 5 min. PCR for each sample was run in a 0.5ml tube using 100ng of total DNA, 10pM of each primer, and 12.5μl master mix (YektaTajhizAzma company, Iran). The presence of PCR product was confirmed by agarose gel electrophoresis (2%).

To evaluate the protective capacities of *B. vulgaris* aqueous extracts on genomic DNA strand breaks, 3μl of extracted DNA (100μg/μl) was mixed with phosphate buffer (H_2_PO_4_, 8.3mM, pH 7.4), and variable concentrations of H_2_O_2_ (3, 5 and 8mM) and FeSO_4_ 0.33mM and EDTA 0.62mM in a final volume of 20μl and were incubated for 120min at 37°C. Next, 3μl of extracted DNA (100ng/μl) was used as DNA protection control. To assess antioxidant capacity of *B. vulgaris *against DNA nicking, various concentrations of *B. vulgaris* extract (0.25, 0.5, 0.75 and 1.0mg/ml) were used. Quantification of PCR product was done by gel electrophoresis-based Polymerase Chain Reaction Method using GelQuant. NET software version 1.8.2.

**Statistical analysis**

In the DNA assays, 15% in quantifications was found as average error. The results were expressed as mean±SD. All tests were done in triplicate. For DNA assays, the two-tailed Mann–Whitney U test at p<0.05 to indicate significant differences between positive control and extracts.

## Results

**DPPH assay**

In DPPH assay, various concentrations (1.25, 2.5 and 5mg/ml) of aqueous extract of *Berberis* showed free radical scavenging activity (21.05, 44.42 and 69.56%, respectively). The Results obtained were comparable to ascorbic acid used as a control.

**Effect of extracts of** ***B. vulgaris*** **against the Fenton-Type oxidative cleavage of** ***pBR322*** **plasmid**

**Figure 1:**
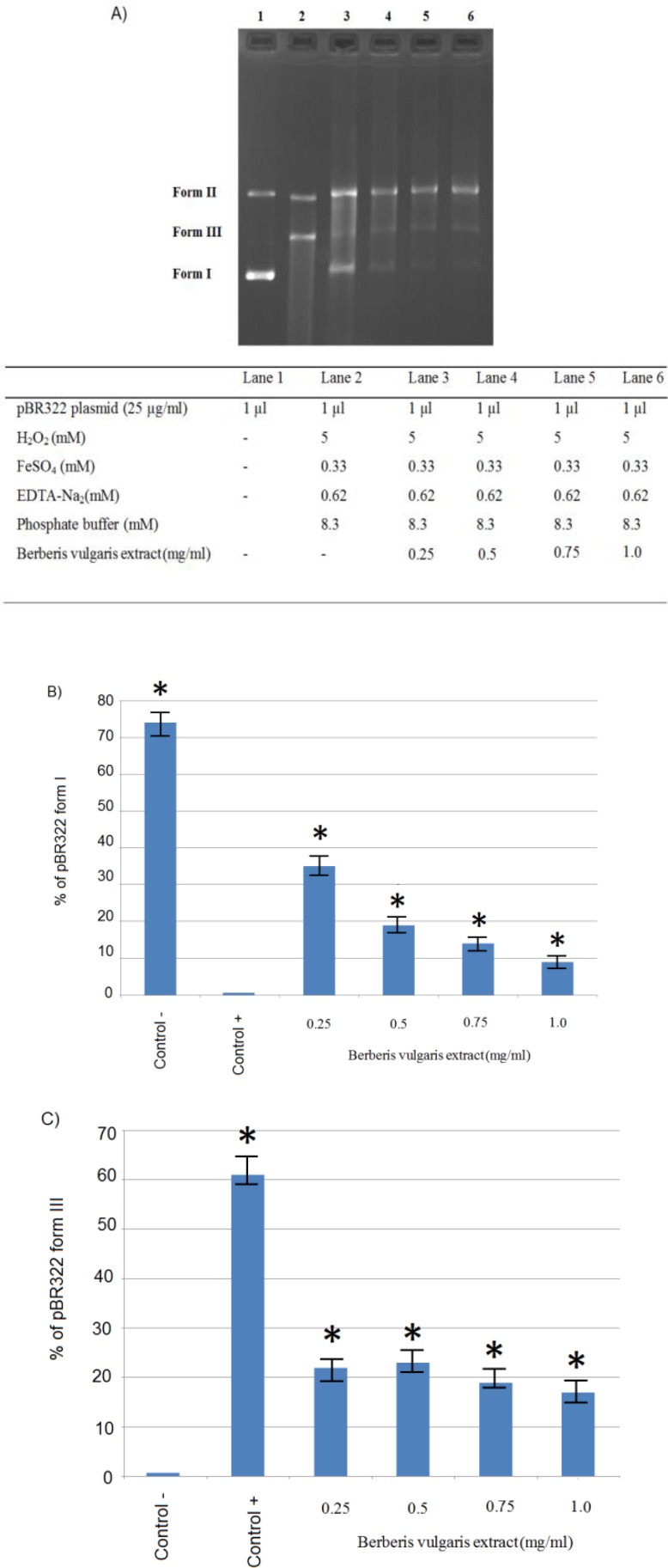
A) Strand breaks protective capacity of *B. vulgaris* extract; B) Quantification of *pBR322* form I protection; C) Quantification of *pBR322* form III formation. All the reaction mixtures were incubated for 20 min at 37°C.* show significant differences between control+ and the other treatments at p<0.05. Form I-supercoiled double stranded DNA; Form II- open circular DNA; and Form III- nicked linear DNA

The *pBR322* plasmid DNA has three forms on agarose gel electrophoresis namely, supercoiled circular DNA (Form I), open circular (Form II), and linear (Form III). Here, we looked for a condition that the *pBR322 *supercoiled circular (form I) was degraded and then protected by adding *B. vulgaris* extract as an antioxidant compound. In this approach, final concentrations of 0.33mM of FeSO_4_ and 0.62mM of EDTA were identified as optimal conditions to evaluate *B. vulgaris* extract •OH nicking protection capacity (lanes 3–6, Figure 1). Our results showed that* B. vulgaris* extract presented dual behavior with a potent antioxidant activity at 1µl (0.25mg/ml) (lane 3), but a pro-oxidant activity at higher concentrations of 2-4µl (0.5-1.0mg/ml) (lanes 4-6).

**Effect of** ***B. vulgaris*** **extract against the Fenton-type oxidative cleavage of genomic DNA**

**Scheme 1:**
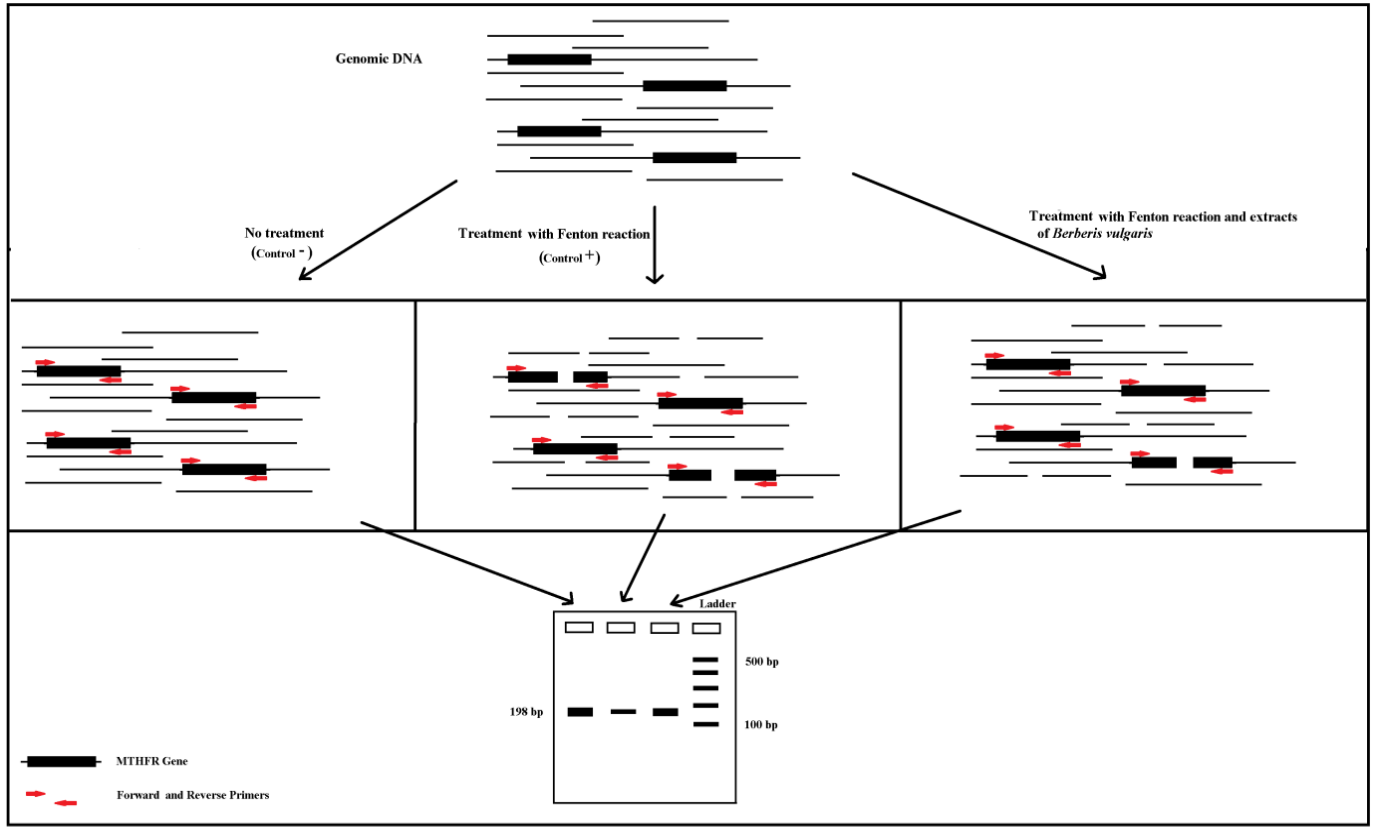
Schematic representation of PCR method to assay of the effect of Fenton reaction on genomic DNA and protective property of *B. vulgaris* extract

PCR method was used to investigate the effect of Fenton reaction on genomic DNA. Schematic representation of PCR method to assay of the effect of Fenton reaction on genomic DNA and protective property of *B. vulgaris* extract is shown in Scheme 1. Cutting the genome DNA by Fenton reaction reduces the number of *MTHFR* template, resulting in lower band intensity in the gel electrophoresis. But, addition of the Berberis extract inhibited the Fenton reaction and prevented DNA damage, resulting in decreased numbers of disrupted *MTHFR* template, and higher PCR product band intensity in the electrophoresis gel.

**Figure 2:**
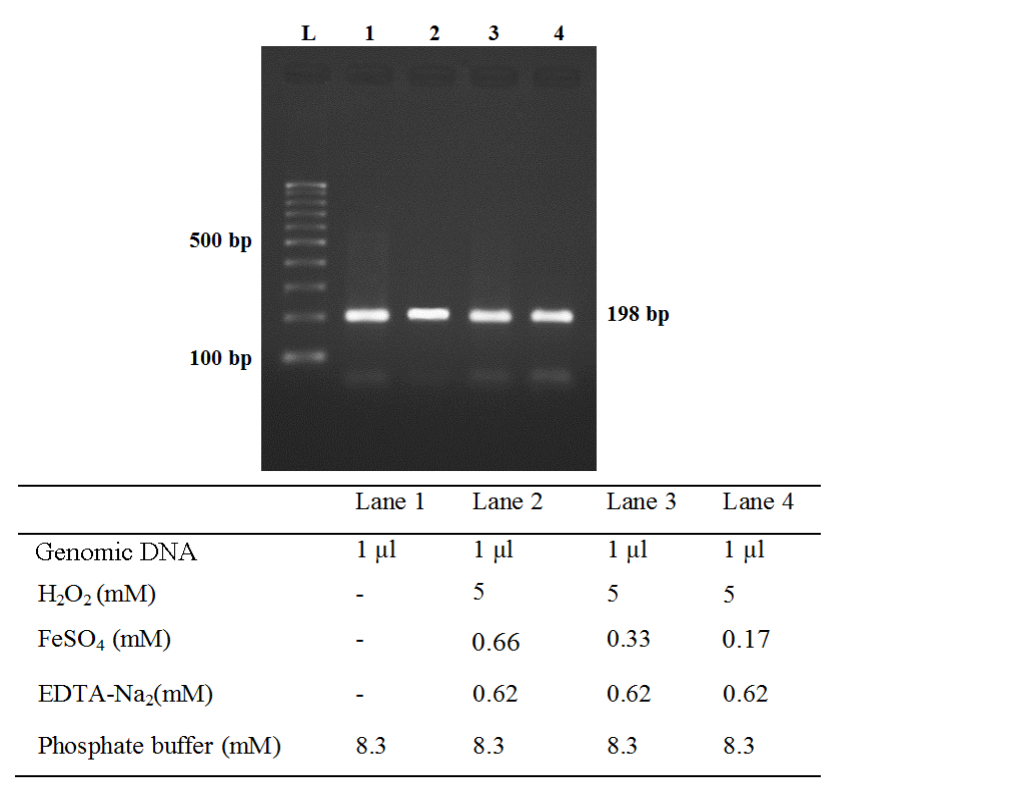
Effect of Fenton reaction on genomic DNA. Quantification of PCR products (after 30 cycles) indicated no significant differences between control assay (Lane 1) and variable concentrations of FeSO_4_ (Lanes 2, 3 and 4)

**Figure 3:**
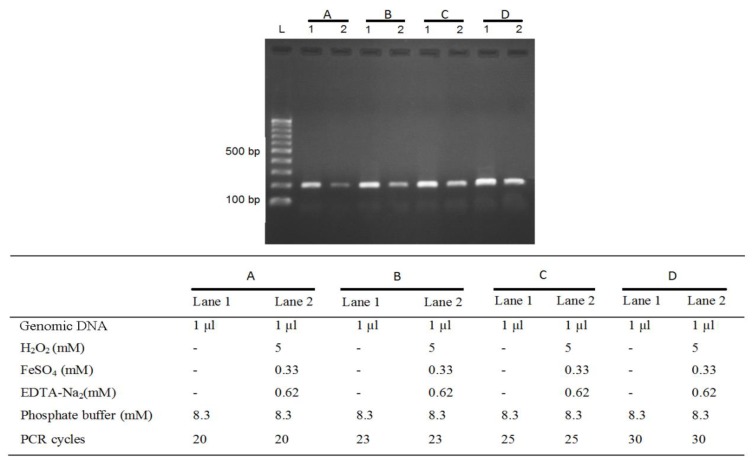
The effect of PCR cycle number on the intensity of PCR bands. Lanes 1 and 2 are amplification of total DNA without and with Fenton reaction, respectively. A) 20, B) 23, C) 25 and D) 30 PCR cycle numbers

The amount of PCR product in a plateau level for all of concentrations of FeSO_4_ was the same (Figure 2). Regardless of the initial amount of FeSO_4_, the amount of amplified products after a sufficient number of PCR cycles were nearly the same. Therefore, to compare the effects of various concentrations of FeSO_4_ on the amount of primary DNA, we described a simple method for measuring the amount of intact genomic DNA with the intensity of the PCR product in variable cycle numbers (20, 23, 25 and 30 cycles, Figure 3). Quantification of PCR products for various cycle numbers showed that 20 cycles provided the optimum conditions for quantification of total DNA without and with Fenton reaction.

**Figure 4:**
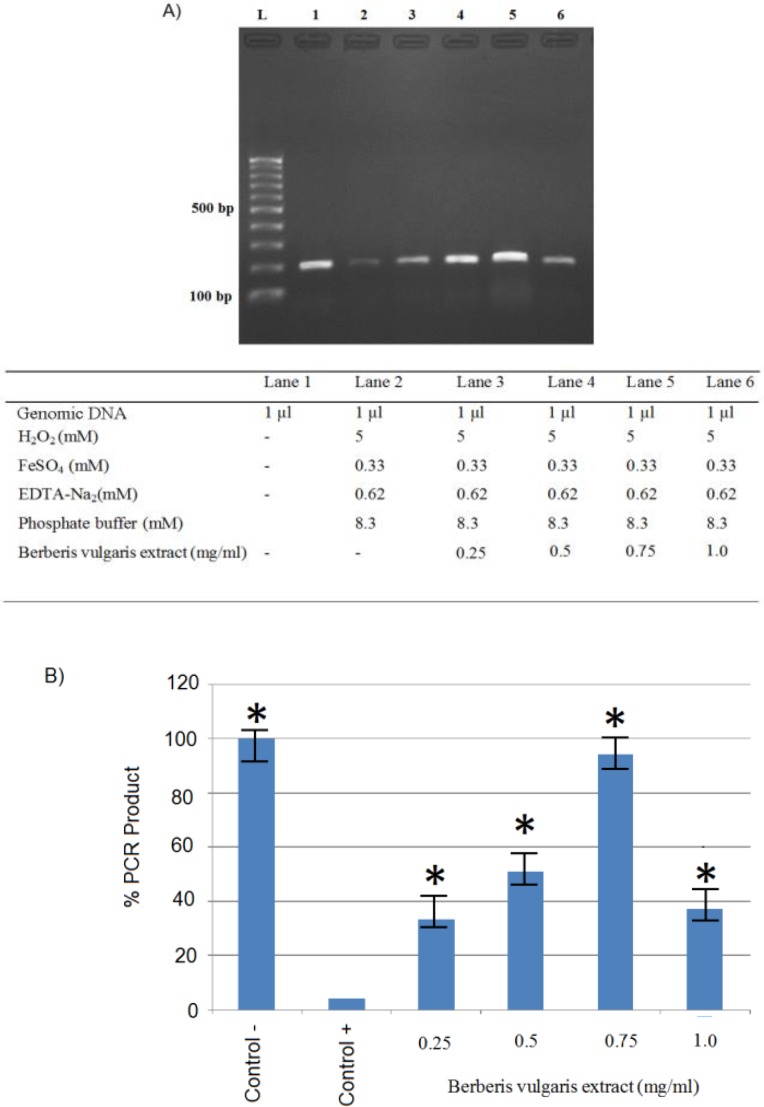
A) PCR products (with genomic DNA breaks) and protective effect of *Berberis vulgaris* extract samples; B) Quantification of extracted DNA protection. * show significant differences between control+ and the other treatments at p<0.05. Reaction mixtures were incubated for 120 min at 37°C

In our study, final concentrations of 0.33mM of FeSO_4_ and 0.62mM of EDTA and 20 cycles of PCR were identified as optimal conditions to evaluate nicking protection capacity of *B. vulgaris* extract on genomic DNA (lanes 3-6, Figure 4).* B. vulgaris* extract presented dual behavior with a potent antioxidant activity at0.25-0.75mg/ml (lanes 3-5, Figure 4), but a pro-oxidant activity at higher concentrations of 1.0mg/ml (lane 6, Figure 4). All reaction mixtures were incubated for 120 min at 37°C. 

## Discussion

Fruits are a natural source of antioxidants. Anthocyanin and polyphenol compounds can reduce the risk of some diseases caused by oxidative stress, such as cardiovascular diseases, degenerative diseases and cancers. Hydrogen peroxide is a prominent factor in ROS production that causes DNA damage in cells (Imlay et al., 1988 ▶; Halliwell and Aruoma, 1991 ▶).

As a medicinal plant,* B. vulgaris* possesses antioxidant and anti-proliferative activities (19, 26). In this study, the antioxidant properties of aqueous extracts of *Berberis* were examined against DNA damage. Our results revealed that the extract of *Berberis* inhibits the Fe_2_^+^- H_2_O_2_-EDTA (Fenton reaction)-induced DNA damage. The *B. vulgaris *rendered protection either by neutralizing H_2_O_2_ or scavenging the ^.^OH generated from the Fenton’s reaction.

Flemmig and Arnhold show that iron in Fenton reaction can mediate DNA strand breaks(Flemmig and Arnhold, 2007 ▶; Mazloum-Ardakani et al., 2013 ▶). Consequently, to determine working conditions that DNA damage is induced by Fenton’s reaction, we evaluated variable concentrations of FeSO_4_.Abbas et al. showed that different cultivars of sugar cane are rich sources of antioxidants and they effectively protected DNA degradation (Abbas et al., 2014 ▶). The extract of* Koelreuteria paniculata* (Sapindaceae) leaves significantly protected DNA degradation induced by Fenton’s reaction in *pUC18* DNA (Kumar et al., 2011 ▶).

Since the Fenton reaction randomly caused single strand or double-stranded DNA breaks, depending on the number of intact DNA molecules, the results were determined by semi-quantitative PCR. An appropriate number of cycles were determined by testing different cycles of 20, 23, 25, and 30. The 20 cycles was selected based on our experiments. The increased concentrations of *B. vulgaris* extract used in the reaction led to higher DNA damage protection(Figure 4; lanes3, 4 and 5) but higher concentrations led to pro-oxidant activities (Bouayed and Bohn, 2010 ▶) (Figure 4; lane 6).

Evaluation of *Berberis* extract activity against *pBR322* plasmid DNA damage indicated that in the presence of the lowest concentration of antioxidant in comparison to genomic DNA, maximum inhibition of the Fenton reaction was observed (0.25vs. 0.75mg/ml). This finding was possibly due to the supercoiled structure of plasmid, as well as its circular and smaller DNA (Ohashi et al., 2002 ▶). 

At higher concentrations, there was an indirect relationship between the concentration and protection rates of extracts of *Berberis* against pBR322 plasmid and genomic DNA damage for the following reasons: First, the best pH for Fenton’s reaction is approximately 3-5 and in acidic conditions, H_2_O_2 _is more protected. With increasing concentration of barberry juice, pH was changed gradually from 7.4 to the acidic pH(Chang et al., 2008 ▶; Jung et al., 2009 ▶).Second, pH is an important factor in moderating the generation of ROS by polyphenols and polyphenolic compounds exert pro-oxidant effect under acidic conditions and high levels of oxygen which leads to higher rates of DNA damage (Ferretti et al., 2010 ▶). All experiments in this study were also performed in the presence of atmospheric oxygen. Third, previous studies showed that polyphenols can directly bind and reduce Fe^3+^ to regenerate Fe^2+^ which will then produce higher levels of hydroxyl radicals (Ohashi et al., 2002 ▶; Sakihama et al., 2002 ▶; Ryan and Hynes, 2007 ▶; Procházková et al., 2011 ▶).

The results indicated that the antioxidant activity of *B. vulgaris* extract can inhibit the production of free radicals and DNA damage. Oxidative cleavage of double-stranded DNA assay is a powerful technique that can be used to determine the antioxidant and pro-oxidant properties of a compound on cellular components such as DNA.
